# Effects of Fixed-Speed Treadmill Exercise on Oxidative Stress and Sirtuin Biomarkers in Young and D-Galactose-Treated Rats: A Biochemical Study with Exploratory Hippocampal Observations

**DOI:** 10.3390/biom16101421

**Published:** 2026-10-01

**Authors:** Beyza Ertan, Yıldırım Gökhan Gencer

**Affiliations:** 1 Department of Coaching Education, Faculty of Sport Sciences, Van Yüzüncü Yıl University, 65090 Van, Türkiye; 2 Department of Physical Education and Sports, Faculty of Sport Sciences, Mersin University, 33110 Mersin, Türkiye; ygokhangencer@mersin.edu.tr

**Keywords:** brain tissue, exercise, molecular aging, exploratory hippocampal histology, oxidative stress

## Abstract

Exercise is proposed to influence oxidative stress and mitochondrial-biogenesis pathways relevant to brain aging, but how exercise intensity relates to these markers alongside hippocampal structure remains unclear. This study examined treadmill exercise at three fixed speeds (15, 20, 30 m/min), corresponding to literature-derived, non-individually validated nominal intensity classifications (~40–50%, 55–65%, 70–75% VO_2_max), on biochemical markers (PGC-1α, MDA, SIRT2, BDNF, irisin) and, as a secondary outcome, hippocampal histology, in young rats and in rats with D-galactose-induced accelerated aging. Sixty-four male Wistar Albino rats (3 months old, 220–350 g) were assigned to control and low-, moderate-, and high-intensity exercise groups within each model category (*n* = 8/group); after six weeks, serum was collected for biochemical analyses and brain tissue was collected for exploratory histological evaluation. In young rats, MDA and SIRT2 differed significantly among groups (*p* < 0.05); PGC-1α, BDNF, and irisin did not. In D-galactose-treated rats, none of the five markers differed significantly; MDA and SIRT2 showed large effect sizes (ε^2^ = 0.207 and 0.199) despite non-significant *p*-values; given the small sample size, this should be read as an imprecise estimate consistent with a range of true effects, including no effect, rather than as evidence of a masked effect. Qualitative, single-rater H&E staining suggested greater dentate gyrus granule-cell density in exercised D-galactose-treated rats versus controls; without neuronal, neurogenesis, or glial markers, this remains a preliminary, hypothesis-generating impression rather than confirmed evidence of neurogenesis. Overall, treadmill exercise was associated with significant changes in oxidative-stress and sirtuin markers across nominal exercise intensities in young rats, the study’s primary contribution; the histological observation requires confirmation with quantitative stereology, specific immunomarkers, and behavioral testing before mechanistic claims can be made.

## 1. Introduction

Aging is a multifactorial process marked by cellular DNA damage, mitochondrial dysfunction, oxidative stress, chronic low-grade inflammation, and progressive decline in musculoskeletal, cardiovascular, and cognitive function [1,2]. Physical exercise is among the most accessible interventions shown to counteract several of these processes; regular activity is associated with improved cognitive function and larger hippocampal volume in older adults [3,4], reduced risk of neurodegenerative disease [5,6], and favorable changes in hippocampal vascularization and mitochondrial biogenesis [7,8,9].

Several circulating and tissue markers are commonly used to index these exercise-related pathways: PGC-1α (mitochondrial biogenesis and antioxidant defense; [10,11]), SIRT2 (NAD^+^-dependent deacetylase linked to metabolic stress response and longevity signaling; [12]), BDNF (synaptic plasticity and neurogenesis via TrkB/PI3K-Akt/MAPK signaling; [13]), irisin (an FNDC5-derived myokine reported to cross the blood–brain barrier and upregulate hippocampal BDNF via a PGC-1α-dependent mechanism; [14,15]), and MDA (a standard marker of lipid peroxidation/oxidative stress). We measured all five in serum in this study, with the important caveat—expanded in the Discussion Section—that peripheral concentrations of these markers do not necessarily reflect hippocampal tissue levels.

The optimal exercise intensity for supporting these pathways has not been established, and responses may differ with age. We used chronic D-galactose administration, a widely used model that induces several features of accelerated aging—oxidative stress, cognitive impairment, and hippocampal neurodegeneration [16,17]—while explicitly acknowledging (see Section 2.2) that it does not reproduce chronological/natural aging in full.

This study examined the effects of treadmill exercise at three nominal intensities on PGC-1α, SIRT2, BDNF, irisin, MDA, and dentate gyrus granule-cell morphology in young and D-galactose-treated rats. We hypothesized that exercise would differentially affect these markers by intensity and by model group, and that D-galactose-treated rats would show a distinct pattern of response compared with young rats.

## 2. Materials and Methods

### 2.1. Experimental Design and Animals

Three-month-old male Wistar Albino rats (initial body weight: 220–350 g) were obtained from the Experimental Animals Laboratory of Van Yüzüncü Yıl University. Only male rats were used to eliminate potential confounding effects of hormonal fluctuations related to the estrous cycle on the measured outcomes. The rats were maintained in a standard laboratory environment (temperature: 22 ± 2 °C, relative humidity: 55% ± 5%, and a 12/12 h light/dark cycle). The experiment was conducted at the Experimental Research and Application Center of Van Yüzüncü Yıl University. This study was approved by the Van Yüzüncü Yıl University Local Ethics Committee for Animal Experiments (Protocol No: 2022/11-07, date: 3 November 2022) and supported by the Scientific Research Projects Unit of Mersin University (Project No: 2023-2-TP3-4927). All experiments were performed in accordance with internationally accepted standard ethical guidelines for the use and care of laboratory animals, as described in the European Community directives (Council Directive 86/609/EEC, 24 November 1986). Throughout the experimental period, all rats had ad libitum access to the same type and quality of standard laboratory chow and water. Individual food intake was not recorded; this is noted as a limitation in Section 4, since differences in energy intake cannot be excluded as a contributor to the body-weight differences observed between groups.

A sample-size calculation was performed prior to the study using G*Power, with the study by Pancar et al. [18] used as a reference and a one-way ANOVA design (α = 5%, power = 95%); this indicated a minimum of 56 animals (7 per group). The specific effect size and other input parameters used in that original calculation were not recorded at the time and cannot now be reliably reconstructed; this is noted as a limitation of the study (see Section 4). One additional animal was added per group during allocation to allow for possible attrition, resulting in groups of 8 rats each; because the original effect-size assumption is unavailable, this addition cannot be shown to confer a specific, quantifiable margin beyond the calculated minimum. A total of 64 male Wistar Albino rats (3 months old, 220–350 g body weight) were included in the study.

Due to the time and financial constraints of obtaining older rats, an induced aging model was created in 3-month-old rats.

Sixty-four male Wistar Albino rats (3 months old at the start of the experiment) were randomly assigned to eight experimental groups using a computer-generated randomization sequence:

Young Groups (*n* = 32):

- Young Control (*n* = 8): No D-galactose, no exercise intervention.
- Young Low-Intensity Exercise (*n* = 8): No D-galactose, treadmill exercise at a nominal 40–50% VO_2_max (fixed speed: 15 m/min).
- Young Moderate-Intensity Exercise (*n* = 8): No D-galactose, treadmill exercise at a nominal 55–65% VO_2_max (fixed speed: 20 m/min).
- Young High-Intensity Exercise (*n* = 8): No D-galactose, treadmill exercise at a nominal 70–75% VO_2_max (fixed speed: 30 m/min).

D-galactose-treated Groups (*n* = 32):

- D-galactose-treated Control (*n* = 8): D-galactose administered, no exercise intervention.
- D-galactose-treated Low-Intensity Exercise (*n* = 8): D-galactose administered, treadmill exercise at 15 m/min.
- D-galactose-treated Moderate-Intensity Exercise (*n* = 8): D-galactose administered, treadmill exercise at 20 m/min.
- D-galactose-treated High-Intensity Exercise (*n* = 8): D-galactose administered, treadmill exercise at 30 m/min.

All rats in the exercise groups underwent the same adaptation and exercise protocols. All rats in the D-galactose-treated groups received the same D-galactose administration protocol.

### 2.2. D-Galactose Induced Aging Model

It is important to clarify that chronic D-galactose administration models specific biochemical and histological features associated with aging—increased oxidative stress, advanced glycation end-product accumulation, and hippocampal neurodegeneration—but is not equivalent to chronological, naturally occurring aging. Throughout this manuscript, we therefore refer to this group as ‘D-galactose-treated’ rather than ‘older’ or ‘aged’, and findings in this group should not be directly extrapolated to natural aging without confirmation in chronologically aged cohorts. Chronic D-galactose administration in rodents has been reported to induce several biochemical and histopathological changes associated with aging [16,19], though the extent to which it replicates natural aging processes remains debated. This accelerated aging model offers several advantages compared to naturally older animals, including:

- Standardization of age-related severity among experimental animals.
- Reduced experimental duration compared to natural aging studies.
- Elimination of survival bias present in naturally older cohorts.
- Cost-effectiveness compared to the maintenance of older animal colonies.

D-galactose-induced aging is characterized by multiple aging-related features, including cognitive impairment [20,21,22], neurodegeneration [23], accumulation of advanced glycation end-products (AGEs) [24], increased free radical production, and elevated oxidative stress in brain tissue [17].

In this study, D-galactose (Sigma-Aldrich, St. Louis, MO, USA) was administered to 3-month-old rats for 7 weeks, 5 days per week, once daily at the same time each day, via intraperitoneal injection at a dose of 150 mg/kg/day [25,26,27]. This 7-week D-galactose administration window began at the start of the 1-week treadmill adaptation period and continued through the entire 6-week main exercise protocol (1 + 6 = 7 weeks in total), so that D-galactose administration, adaptation, and exercise training overlapped throughout the study, with tissue and blood collection occurring at the end of week 6 of exercise (i.e., within the ongoing 7-week D-galactose window).

From the first week of D-galactose administration, observable changes in the rats’ fur were noted, which were considered indicative of aging. At the end of the experiment, biochemical and histopathological parameters were examined, revealing findings that supported the aging process. In addition to these external changes in fur, the increased levels of oxidative stress observed in biochemical analyses and the tissue changes detected in histopathological examinations are consistent with, but do not by themselves establish, an aging-like phenotype in this model; as noted above, we therefore refer to this group as “D-galactose-treated” rather than “aged” throughout the manuscript.

This absolute speed approach is consistent with established protocols in D-galactose aging model research. Multiple studies have demonstrated that D-galactose-induced aged rats can successfully complete treadmill protocols at speeds comparable to young controls [28,29,30,31]. The induced aging phenotype in D-galactose models primarily affects metabolic and oxidative parameters rather than physical exercise capacity, which distinguishes this model from naturally aged (24–30 month old) animals that exhibit substantial decrements in exercise tolerance.

### 2.3. Treadmill Exercise Program

Rats in the running groups underwent treadmill exercise for 6 weeks, 5 days per week. The exercises were conducted using a 6-lane custom treadmill system (MAT-TIME 0804; Commat Ltd., Ankara, Türkiye). Prior to the start of the exercise program, all rats underwent a 1-week adaptation period. During the adaptation phase, the rats ran at a speed of 5 m/min for progressively increasing durations: 10 min on day 1, 12 min on day 2, 15 min on day 3, 17 min on day 4, and 20 min on day 5. Animals that consistently showed reluctance to run (defined a priori as failure to maintain pace for more than 50% of the session duration on two consecutive adaptation days) or exhibited signs of significant stress during this phase were excluded and replaced (total *n* = 3): one from the young low-intensity exercise group, one from the young moderate-intensity exercise group, and one from the D-galactose-treated low-intensity exercise group. Replacement animals were drawn from the same source population and assigned following the same computer-generated randomization procedure used for the original allocation, ensuring that group assignment was not influenced by the exclusion event itself.

Following the adaptation period, the experimental exercise speeds were set according to the exercise intensity classification defined by Wang et al. [32]. This classification is based on the relationship between treadmill speed and oxygen consumption (VO_2_) in rats, as reported by Bedford et al. [33]. Accordingly, low-, moderate-, and high-intensity exercises corresponded to speeds of 15, 20, and 30 m/min, respectively, representing approximately 40–50%, 55–65%, and 70–75% of VO_2_max as nominal, literature-derived classifications (see qualification below). These speed ranges are consistent with standard protocols commonly used in experimental exercise physiology studies. All exercise sessions, during both the adaptation period and the 6-week main protocol, were conducted between 08:00 and 12:00 to minimize circadian variability. During the 6-week main exercise period, each session lasted 20 min/day for the low-, moderate-, and high-intensity groups alike, 5 days/week. No separate warm-up or cool-down beyond the 1-week adaptation protocol described above was used.

The same absolute running speeds were applied to young and D-galactose-treated rats to allow direct speed comparability. We emphasize that the 40–50%, 55–65%, and 70–75% VO_2_max ranges cited above are literature-derived reference values [32,33], not individually measured physiological intensities—VO_2_max, blood lactate, and heart rate were not measured in this study. Because aerobic capacity may be reduced in the D-galactose model, the same absolute speed likely corresponded to a different relative physiological intensity in the two groups. We therefore describe our exercise groups as differing in nominal/assigned intensity, and we avoid describing our results as demonstrating true physiological ‘intensity-dependent’ effects without this qualification. We acknowledge this as a methodological limitation; future studies should incorporate individualized intensity calibration through maximal exercise testing.

Throughout each exercise session, the animals were continuously monitored by trained research personnel. No signs of fatigue, reluctance to run, or abnormal behaviors were observed. Electrical stimulation was not required, and all animals adapted naturally to the prescribed exercise program. No rat exhibited stress or exertion requiring session termination at any point during the study. All rats fully adhered to the exercise protocol, and no session losses occurred.

Our treadmill system (MAT-TIME 0804; Commat Ltd., Ankara, Türkiye) was equipped with an electrical stimulation grid; however, this feature was not activated during any phase of the study. Instead, exercise compliance was achieved through comprehensive habituation training and, when necessary, gentle manual guidance techniques as described by Khataei et al. [34] and recommended in recent American Physiological Society guidelines. This approach was deliberately chosen to avoid the potential confounding effects of electrical shock-induced stress on the neurobiological and behavioral parameters under investigation. The 1-week progressive adaptation protocol allowed all rats in the final exercise groups to complete sessions without requiring aversive stimulation. Three animals that showed persistent reluctance to run during the adaptation phase were excluded and replaced to ensure data quality. We acknowledge that our compliance rate and lack of electrical stimulation requirement may be partly attributed to the use of D-galactose-induced aging rather than naturally aged (24–30 month old) animals, as the former model preserves greater physical exercise capacity despite inducing metabolic and cognitive aging phenotypes.

### 2.4. Biochemical Analysis

The rats were anesthetized via intraperitoneal injection of 75 mg/kg ketamine (Ketalar®; Pfizer, İstanbul, Türkiye) and 10 mg/kg xylazine (Rompun®; Bayer, İstanbul, Türkiye). At the end of the 6-week experimental period (1 day after the exercise program), the rats were sacrificed in a sterile environment, and 5 mL of blood was collected using the intracardiac method. Blood samples were collected into serum separator tubes and centrifuged at 4000 rpm for 10 min to separate the serum. The serum was then transferred into pre-assigned, numbered Eppendorf tubes and stored at −80 °C in a freezer (Sanyo Electric Co., Ltd., Osaka, Japan) until the day of analysis.

Serum concentrations of MDA, SIRT2, BDNF, PGC-1α, and irisin were measured using commercially available enzyme-linked immunosorbent assay (ELISA) kits supplied by Wuhan Fine Biological Technology Co. (Wuhan, China) at the Biochemistry Laboratory of Van Yüzüncü Yıl University. The following individually branded kits were used: USCN Rat MDA (Malondialdehyde) ELISA Kit (Catalog No: CEA597Ge); USCN Rat SIRT2 (Sirtuin 2) ELISA Kit (Catalog No: SEA430Ra); USCN Rat BDNF (Brain-Derived Neurotrophic Factor) ELISA Kit (Catalog No: SEA011Ra); USCN Rat FNDC5/Irisin ELISA Kit (Catalog No: SEN576Ra); and SUNLONG BIOTECH Rat PGC-1α ELISA Kit (Catalog No: SL0828Ra). The manufacturer-reported standard-curve detection ranges were as follows: MDA, 24.69–2000 ng/mL; SIRT2, 1.56–100 ng/mL; BDNF, 31.2–2000 pg/mL; irisin/FNDC5, 31.2–2000 ng/mL; PGC-1α, 40–480 pg/mL. Standards and samples were serially diluted and processed according to the manufacturer’s protocol (sequential incubation with detection reagents at 37 °C, followed by substrate development and absorbance reading at 450 nm). All procedures were performed according to the manufacturer’s instructions. Absorbance was measured at 450 nm using a microplate reader (ELx800; BioTek Instruments, Inc., Winooski, VT, USA), and concentrations were calculated from standard curves generated for each analyte. Samples were assayed in singlicate (one well per sample) due to serum volume constraints. The manufacturer-reported intra-assay and inter-assay coefficients of variation were <10% and <12%, respectively; because samples were not run in replicate within this experiment, an experiment-specific intra-assay CV could not be calculated, and this is noted as a limitation. Samples underwent a single freeze–thaw cycle, and hemolyzed samples were excluded prior to analysis.

### 2.5. Qualitative Histomorphological Evaluation

Histopathological evaluations in the study were performed using services provided by the Histology Laboratory of Van Yüzüncü Yıl University. The histological analysis in this study was qualitative; no stereological cell counting, unbiased sampling grid, or automated quantification was performed. The findings below should be read as descriptive observations rather than quantitatively confirmed group differences. After sacrifice, tissue samples were collected from the cornu ammonis region of the rats’ brains and fixed in 10% neutral buffered formalin solution. Following an automatic tissue processing procedure, the samples were embedded in paraffin, and 4 μm-thick sections were cut from the paraffin blocks using a rotary microtome (RM2235; Leica Biosystems, Nussloch, Germany). For each animal, three to five coronal sections were examined at the level of the dorsal hippocampus, corresponding approximately to Bregma −3.30 mm to −4.16 mm (Paxinos & Watson rat brain atlas), consistent with the standard sampling levels used in comparable D-galactose/exercise hippocampal histology studies. The most representative section for each animal, showing clear CA1–CA4 and dentate gyrus orientation without processing artifact, was selected for photomicrography. The sections were stained with hematoxylin–eosin and examined under a light microscope (Eclipse 80i; Nikon Corporation, Tokyo, Japan), with the observed morphological changes documented using a digital camera (DS-Ri2; Nikon Corporation, Tokyo, Japan). During the histopathological examination, morphological changes in the cornu ammonis region of the brain were evaluated in terms of regeneration, inflammation (cellular infiltration, gliosis), and degenerative–necrotic alterations, and photographic records were obtained.

Histopathological evaluations were conducted by an experienced veterinary pathologist with over 10 years of expertise in rodent neuropathology. The evaluator was blinded to group assignments during the assessment. We acknowledge that quantitative stereological methods and neurogenesis-specific markers (e.g., DCX, Ki67, BrdU, NeuN) were not employed in this study; therefore, histopathological findings are described qualitatively, and the term ‘neuroregeneration’ should be interpreted with caution as reflecting morphological changes in granule cell density rather than confirmed neurogenesis.

### 2.6. Statistical Analysis

The data analysis for this study was performed using SPSS 25.0. The sample size calculation for the study was conducted using the G*Power program (version 3.1.9.2). The normality of all data was assessed using the Shapiro-Wilk test. For data showing a parametric distribution, one-way analysis of variance (ANOVA) was applied. The homogeneity of variances was examined using Levene’s test. Differences in group means for the parametric variables MDA and PGC-1α in the young group and PGC-1α in the D-galactose-treated group were analyzed using one-way ANOVA. Arithmetic means and standard deviations were used for continuous variables. For nonparametric variables, the Kruskal–Wallis H test was applied. To determine which group(s) contributed to significant differences, post hoc analysis using Dunn’s test with Bonferroni adjustment for pairwise comparisons was performed. To assess whether the effects of exercise intensity differed between model groups, a two-way ANOVA (model × exercise condition) was performed for PGC-1α, for which residuals met the normality assumption (Shapiro-Wilk *p* > 0.05); for MDA, SIRT2, BDNF, and irisin, whose model-wise distributions or two-way residuals departed from normality (Section 3.1), the Scheirer–Ray–Hare test—a rank-based, nonparametric analog of the two-way ANOVA—was used instead, to test the main effects of model and exercise condition and their interaction. Homogeneity of variances across the eight model × exercise cells was assessed using Levene’s test (Section 3.1). Because body weight was recorded repeatedly in the same animals (weeks 0, 3, and 6), it was analyzed separately from the single-timepoint biochemical markers using a linear mixed-effects model with time, model, exercise condition, and their interactions as fixed effects and a random intercept and slope for time by animal; fixed effects were evaluated by likelihood-ratio tests comparing nested maximum-likelihood fits (Section 3.2). Effect sizes were calculated for all comparisons: partial eta-squared (η^2^) for one-way ANOVA, and epsilon-squared (ε^2^) for Kruskal–Wallis analyses, following conventional benchmarks (small: 0.01, medium: 0.06, large: 0.14). A significance level of *p* < 0.05 was considered statistically significant. Additionally, 95% confidence intervals for group means (parametric variables, Table 1) were calculated from the reported mean, SD, and n = 8/group using the t-distribution (df = 7): Young MDA—Control 90.3 [81.61, 98.99], Low 90.5 [80.89, 100.11], Moderate 103.2 [94.67, 111.73], High 85.9 [78.79, 93.01] ng/mL; Young PGC-1α—Control 47.2 [37.67, 56.73], Low 54.1 [47.16, 61.04], Moderate 53.0 [40.79, 65.21], High 48.5 [28.60, 68.40] pg/mL; and D-galactose-treated PGC-1α—Control 48.9 [34.19, 63.61], Low 44.7 [29.23, 60.17], Moderate 51.4 [40.45, 62.35], High 57.8 [40.41, 75.19] pg/mL. The factorial model × exercise-condition analysis (see Section 3.1, “Model × Exercise Interaction Effects”) was treated as the primary analysis for testing whether exercise responses differ between the young and D-galactose-treated models; within-model one-way comparisons (Tables 1 and 2) are reported as secondary/exploratory analyses. We did not prespecify a single primary biochemical outcome prior to data collection, and we note this as a limitation in Section 4, together with the fact that no correction for multiplicity across the five outcome families (MDA, SIRT2, BDNF, PGC-1α, irisin) was applied beyond the Bonferroni/Dunn correction already used within each outcome’s own pairwise comparisons. All five outcomes are treated with equal exploratory status, and the results should be interpreted accordingly.

## 3. Results

**Figure 1:**
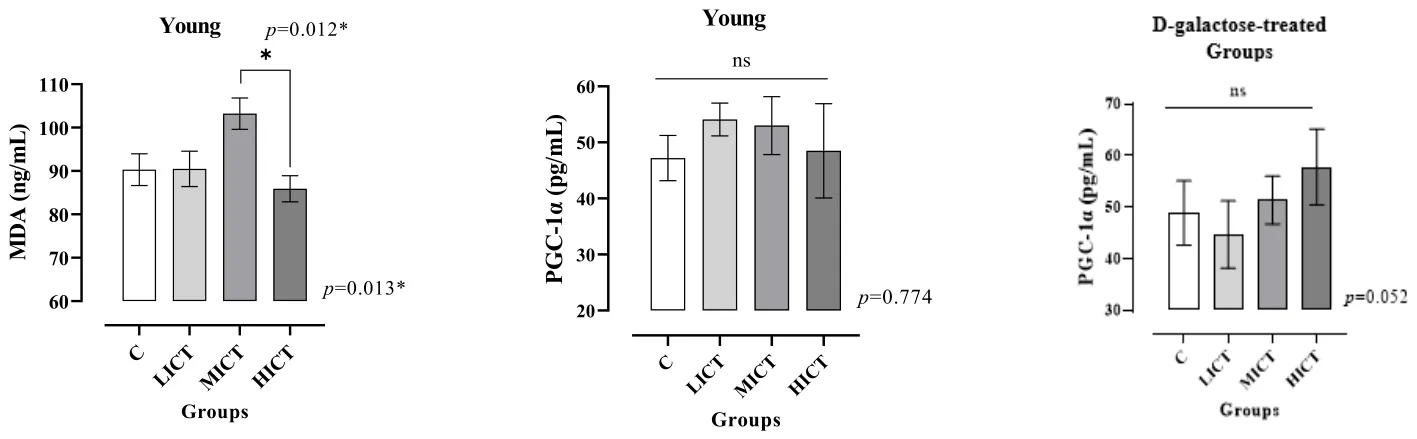
Graph of mean values for MDA and PGC-1α. * *p* < 0.05, ns not significant.

**Figure 2:**
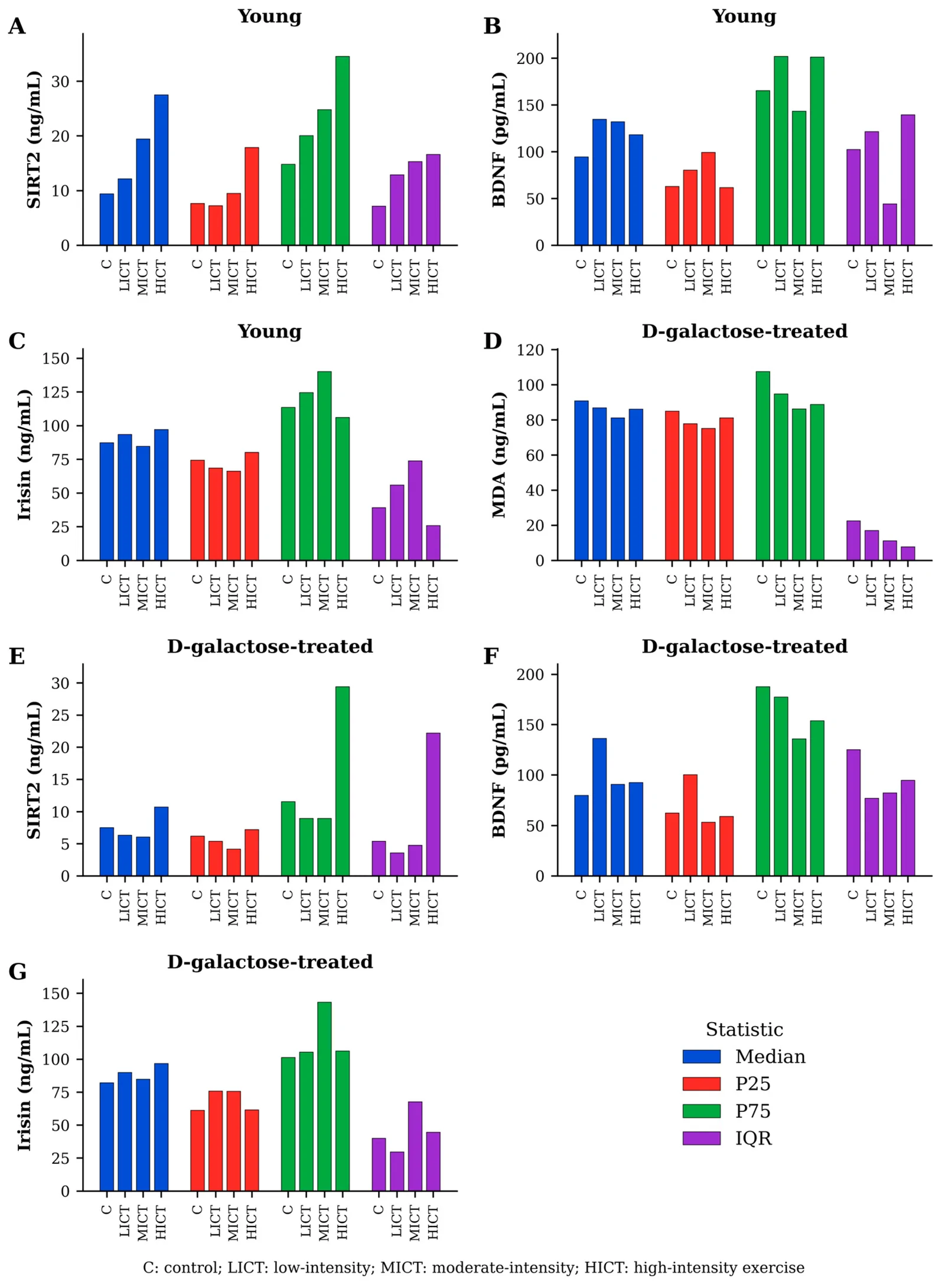
Median, 25th percentile (P25), 75th percentile (P75), and interquartile range (IQR) values of SIRT2, BDNF, and irisin in young rats (**A**–**C**) and of MDA, SIRT2, BDNF, and irisin in D-galactose-treated rats (**D**–**G**).

The results obtained in this study and their statistical analyses are presented in Table 1 and Table 2. In young rats, a statistically significant difference was observed among groups in terms of malondialdehyde (MDA) and sirtuin 2 (SIRT2) levels (*p* < 0.05), whereas no significant difference was found for peroxisome proliferator-activated receptor gamma coactivator 1-alpha (PGC-1α), brain-derived neurotrophic factor (BDNF), and irisin levels (*p* > 0.05). In D-galactose-treated rats, no statistically significant differences were detected among groups for MDA, SIRT2, PGC-1α, BDNF, and irisin levels (*p* > 0.05). The mean values of the variables presented in Table 1 are shown in Figure 1, while the median and percentile values of the variables in Table 2 are illustrated in Figure 2.

The results of this study are organized according to model group (young and D-galactose-treated) and outcome measure (biochemical markers and histopathological findings). Statistical comparisons were performed among exercise intensity groups within each age category. Complete descriptive statistics for all measured variables are presented in Table 1 (parametric data) and Table 2 (non-parametric data), with visual representations shown in Figure 1 and Figure 2.

In young rats, analysis revealed a significant difference among groups in MDA levels [F(3,28) = 4.23, *p* = 0.013, η^2^ = 0.312]; specifically, MDA levels in the young moderate-intensity exercise group (103.2 ± 10.2 ng/mL) were significantly higher compared to the young control group (90.3 ± 10.4 ng/mL; *p* = 0.042) and the young high-intensity exercise group (85.9 ± 8.5 ng/mL; *p* = 0.011). For SIRT2, the Kruskal–Wallis test showed a significant overall difference among groups [H(3) = 8.224, *p* = 0.042, ε^2^ = 0.265]. Dunn’s post hoc pairwise comparisons with Bonferroni adjustment showed that SIRT2 was significantly higher in the high-intensity exercise group than in the control group (adjusted *p* = 0.042). The moderate-intensity versus control comparison was not statistically significant after adjustment (adjusted *p* = 0.922). No statistically significant differences were observed among groups for PGC-1α [F(3,28) = 0.37, *p* = 0.774, η^2^ = 0.038], although higher means were noted in the young low-intensity exercise group (54.1 ± 8.3 pg/mL) and the young moderate-intensity exercise group (53 ± 14.6 pg/mL). Similarly, BDNF levels did not differ significantly [χ^2^(3) = 1.27, *p* = 0.735, ε^2^ = 0.041]; the median values in all exercise groups (young low-intensity: 134.75 pg/mL; young moderate-intensity: 132.03 pg/mL; young high-intensity: 118.3 pg/mL) were numerically higher than in the control group (94.48 pg/mL), but given the non-significant result and small effect size, this numerical difference should not be interpreted as an exercise effect. Likewise, no significant differences were observed for irisin [χ^2^(3) = 0.31, *p* = 0.975, ε^2^ = 0.010], with all groups showing similar median levels (young control: 87.36 ng/mL; young low-intensity: 93.39 ng/mL; young moderate-intensity: 84.61 ng/mL; young high-intensity: 97.05 ng/mL).

In D-galactose-treated rats, MDA did not differ significantly among groups [χ^2^(3) = 6.42, *p* = 0.082, ε^2^ = 0.207]; all exercise groups had numerically lower median MDA than the D-galactose-treated control group (90.88 ng/mL), most notably the moderate-intensity group (81.16 ng/mL). The *p*-value did not cross the significance threshold; given the small sample size, this large effect size should be read as an imprecise estimate consistent with a range of true effects, including no effect, rather than as evidence of a masked effect. SIRT2 levels did not differ significantly [χ^2^(3) = 6.18, *p* = 0.096, ε^2^ = 0.199]; the high-intensity group had a numerically higher median (10.74 ng/mL) than the other exercise groups. As with MDA above, this non-significant, large-effect-size finding should be read as an imprecise estimate rather than a probable masked effect. PGC-1α levels showed no statistically significant differences [F(3,28) = 0.76, *p* = 0.522, η^2^ = 0.075, medium effect]; values were numerically lower at low intensity (44.7 pg/mL) and numerically higher at high intensity (57.8 pg/mL) relative to control. BDNF analysis revealed no significant difference [χ^2^(3) = 2.99, *p* = 0.399, ε^2^ = 0.096]; the median values in the exercise groups (especially low-intensity: 136.2 pg/mL) were numerically higher than in the control group (79.92 pg/mL). Irisin levels did not show significant differences [χ^2^(3) = 0.827, *p* = 0.843, ε^2^ = 0.027]; all exercise groups had numerically higher median values than D-galactose-treated control. These non-significant results are reported for completeness and should not be read as demonstrated exercise effects; the two markers with large effect sizes (MDA, SIRT2) are the strongest candidates for further study with a larger sample.

### 3.1. Model × Exercise Interaction Effects

To directly test whether the effect of exercise condition on each biomarker differed between young and D-galactose-treated rats, we fitted a factorial model × exercise-condition analysis for each biomarker (Table 3 reports the underlying Shapiro–Wilk and Levene diagnostics that determined which method was used). For PGC-1α, whose two-way residuals were approximately normal, a two-way ANOVA showed no significant main effect of model [F(1,56) = 0.00, *p* = 0.999], exercise condition [F(3,56) = 0.32, *p* = 0.809], or model × exercise interaction [F(3,56) = 0.86, *p* = 0.465]. For MDA, SIRT2, BDNF, and irisin, the Scheirer–Ray–Hare test was used, given non-normal residuals. For MDA, this revealed a significant model × exercise interaction [H(3) = 10.45, *p* = 0.015], alongside a significant main effect of model [H(1) = 4.00, *p* = 0.045] and a non-significant main effect of exercise condition [H(3) = 3.94, *p* = 0.268]; this significant interaction indicates that the effect of exercise condition on MDA genuinely differed between young and D-galactose-treated rats, consistent with the pattern already visible in Table 1 and Table 2 (a significant, non-monotonic exercise effect in young rats but no significant exercise effect in D-galactose-treated rats). For SIRT2, both the main effect of model [H(1) = 13.30, *p* < 0.001] and of exercise condition [H(3) = 9.90, *p* = 0.019] were significant, but the model × exercise interaction was not [H(3) = 1.97, *p* = 0.578], indicating that while SIRT2 levels differ overall between models and across exercise conditions, the exercise effect itself did not differ significantly by model. For BDNF [model: H(1) = 0.70, *p* = 0.401; exercise: H(3) = 3.07, *p* = 0.381; interaction: H(3) = 0.95, *p* = 0.814] and irisin [model: H(1) = 0.41, *p* = 0.524; exercise: H(3) = 0.38, *p* = 0.944; interaction: H(3) = 1.04, *p* = 0.792], no main effects or interaction reached significance. Taken together, these factorial analyses support treating MDA as a marker whose exercise response is genuinely model-dependent, while SIRT2’s exercise response, though robust overall, did not differ significantly between models; for BDNF and irisin, the data do not support any model-dependent or exercise-dependent pattern.

### 3.2. Body Weight Changes

Body weight was recorded at week 0 (baseline), week 3, and week 6 for all eight groups (Table 4). In young rats, one-way ANOVA showed significant between-group differences at each time point (week 0: F(3,28) = 5.02, *p* = 0.007; week 3: F(3,28) = 13.10, *p* < 0.001; week 6: F(3,28) = 24.12, *p* < 0.001). In D-galactose-treated rats, body weight did not differ significantly among groups at baseline (F(3,28) = 2.63, *p* = 0.070) but diverged significantly by week 3 (F(3,28) = 3.56, *p* = 0.027) and week 6 (F(3,28) = 30.70, *p* < 0.001), with the D-galactose-treated high-intensity exercise group showing progressive weight loss relative to control. Food intake was not measured during the study; this is noted as a limitation in Section 4, since it could otherwise help interpret these body-weight and metabolic findings. Because body weight was measured repeatedly in the same animals, the separate one-way ANOVAs reported above do not account for within-animal correlation. We therefore fitted a linear mixed-effects model to the full longitudinal dataset (192 observations from 64 animals), with time, model (young vs. D-galactose-treated), exercise condition, and their interactions as fixed effects and a random intercept and slope for time by animal; fixed effects were tested by likelihood-ratio tests comparing nested maximum-likelihood fits. This model showed significant main effects of time (χ^2^(1) = 48.80, *p* < 0.001) and exercise condition (χ^2^(3) = 17.91, *p* = 0.0005), and a significant time × exercise interaction (χ^2^(3) = 70.55, *p* < 0.001), indicating that the weight trajectory over the six weeks differed by exercise intensity, with the high-intensity groups showing markedly attenuated or negative weight gain (Table 4). The main effect of model (χ^2^(1) = 0.32, *p* = 0.573) and the time × model interaction (χ^2^(1) = 1.97, *p* = 0.161) were not significant, indicating a similar overall trajectory shape in young and D-galactose-treated animals. A model × exercise interaction was significant (χ^2^(3) = 20.21, *p* < 0.001); the three-way time × model × exercise interaction was not significant (χ^2^(3) = 5.73, *p* = 0.126) and was dropped from the final model. Because body weight already differed significantly among the young groups at baseline (week 0: F(3,28) = 5.02, *p* = 0.007; Table 4), the model × exercise interaction—which reflects differences in overall weight level rather than in growth rate—cannot be fully separated from this pre-existing imbalance and should not be read as clear evidence of a differential response to exercise between models. The time × exercise interaction, which captures divergence in trajectories after group assignment, is less affected by the baseline imbalance and is interpreted as the more informative test of exercise’s effect on body weight.

### 3.3. Exploratory Qualitative Histomorphological Observations (Secondary Outcome)

Light microscopic examination of brain sections from the young control group (young control) and the young exercise groups (young low-intensity exercise, young moderate-intensity exercise, and young high-intensity exercise) revealed normal histological architecture in the hippocampal regions. The cornu ammonis (CA1–CA4) and dentate gyrus (DG) maintained their characteristic three-layered organization without any signs of neuronal degeneration, inflammatory cell infiltration, or gliosis. Among the four subfields of the cornu ammonis, CA1 and CA2 were composed of three to four layers of small pyramidal cells with vesicular nuclei, while CA3 and CA4 contained larger pyramidal cells with vesicular nuclei. Structurally, the cornu ammonis consisted of three layers from the outermost to the innermost: the molecular layer (M), the pyramidal layer (P), and the polymorphic layer (PO). The molecular layer was characterized by organized neuronal structures, scattered glial cells, and nerve cells.

The dentate gyrus consisted of the molecular cell layer (M), the granular cell layer (G), and the polymorphic cell layer (PO) on both sides of the CA4 region, as well as the suprapyramidal (S) and infrapyramidal (I) blades. The open part of the dentate gyrus displayed a dark V-shaped structure surrounding the CA4 region. No inflammatory infiltration, gliosis, or degenerative–necrotic changes were observed in any of the sections. Comparative evaluation of brain sections from young rats subjected to low-, moderate-, and high-intensity exercise did not reveal significant morphological differences compared to the young control group, indicating that exercise did not induce notable changes in the hippocampal structure or dentate gyrus morphology.

**Figure 3:**
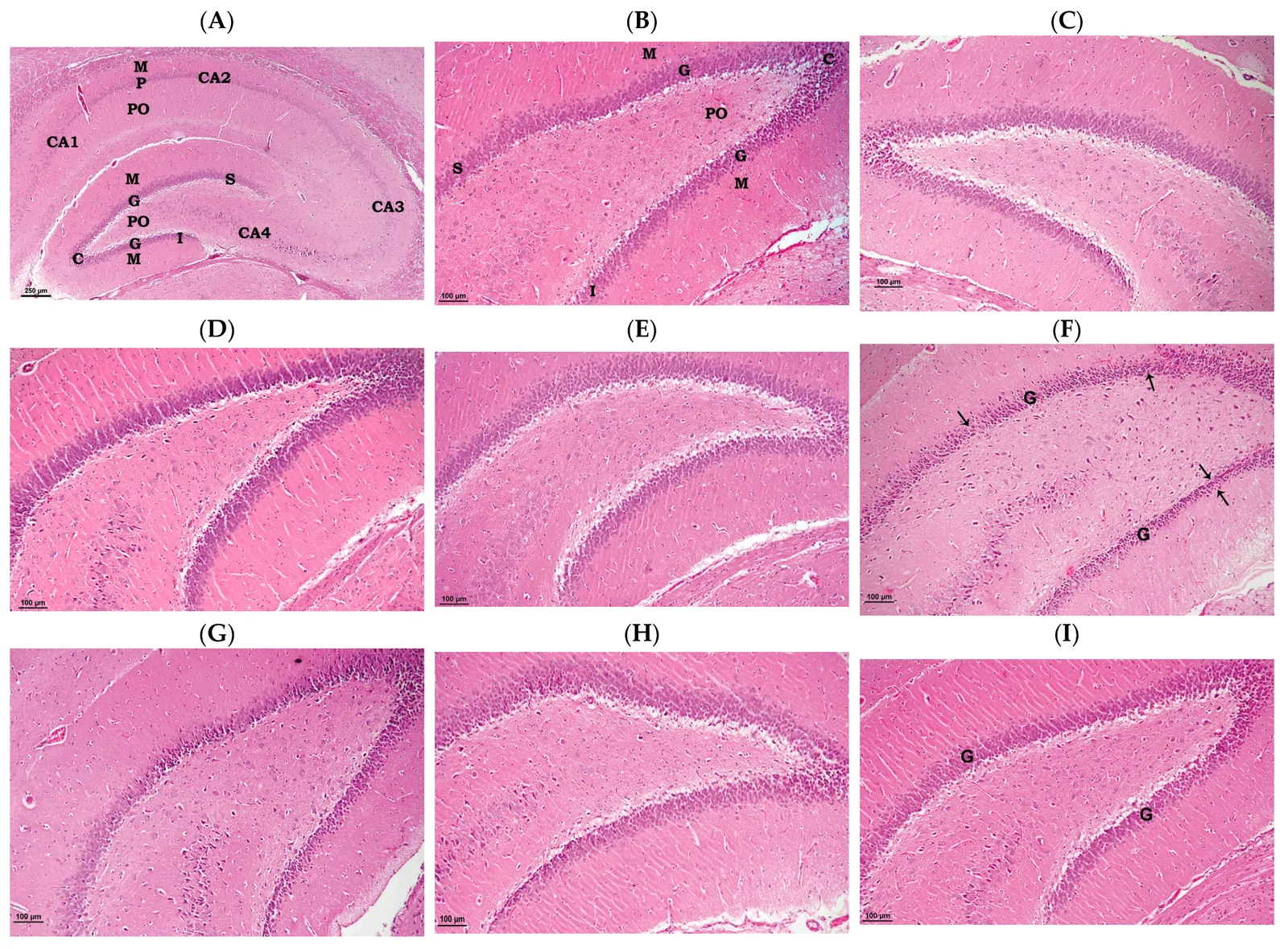
(**A**): Young control group: normal histological appearance of the hippocampus and dentate gyrus. The hippocampus consists of four subfields: CA1, CA2, CA3, and CA4. Each subfield comprises three layers: the molecular layer (M), pyramidal layer (P), and polymorphic layer (PO). The dentate gyrus appears as a dark V-shaped structure surrounding the CA4 region of the hippocampus. It consists of three layers: the granular cell layer (G), molecular layer (M), and polymorphic layer (PO). The suprapyramidal (S) and infrapyramidal (I) blades of the dentate gyrus are shown. The crest (C) represents the junction of both blades. H&E, Bar = 250 µm. (**B**): Young control group, higher magnification of the dentate gyrus shown in (**A**). H&E, Bar = 100 µm. (**C**): Young low-intensity exercise group. H&E, Bar = 100 µm. (**D**): Young moderate-intensity exercise group. H&E, Bar = 100 µm. (**E**): Young high-intensity exercise group. H&E, Bar = 100 µm. (**F**): D-galactose-treated control group: on visual inspection, a qualitatively thinner-appearing granular layer (G) of the dentate gyrus (arrows). H&E, Bar = 100 µm. (**G**): D-galactose-treated low-intensity exercise group. H&E, Bar = 100 µm. (**H**): D-galactose-treated moderate-intensity exercise group. H&E, Bar = 100 µm. (**I**): D-galactose-treated high-intensity exercise group: a qualitatively greater apparent density of cells in the granular layer, based on single-rater visual assessment without quantitative cell counting. H&E, Bar = 100 µm.

In D-galactose-treated control animals, the dentate gyrus granule cell layer appeared visually thinner and more sparsely packed than in young control sections on qualitative inspection (Figure 3F), consistent with the oxidative/neurodegenerative changes reported for this model. In the D-galactose-treated exercise groups, qualitative examination by the blinded pathologist suggested a greater density/packing of dentate gyrus granule cells compared with the D-galactose-treated control group (Figure 3G,H), with the high-intensity group showing the most pronounced visual difference in granular-layer cell density (Figure 3I). This is reported as a preliminary, descriptive, qualitative impression; it was not quantified (no cell counts, no stereology), was assessed by a single rater, and should not be interpreted as confirmed evidence of increased cell number, neurogenesis, or regeneration.

## 4. Discussion

In this study, the effects of treadmill exercise at three nominal intensities on MDA, SIRT2, BDNF, PGC-1α, and irisin levels, as well as hippocampal histology, were investigated in young rats and rats subjected to D-galactose-induced accelerated aging. The D-galactose model was associated with histopathological changes characterized by a qualitatively reduced number of dentate gyrus granular cells, although serum biochemical markers did not fully reflect this pattern. Seven weeks of D-galactose administration (150 mg/kg/day) preceded histopathological changes that were consistent with those reported in this model, including a visibly thinner dentate gyrus granular cell layer. However, no significant differences in serum biochemical markers were observed between young and D-galactose-treated control groups, and PGC-1α levels did not show the age-related decline reported elsewhere in the natural aging literature [35,36] (Table 1 and Table 2). This may reflect the D-galactose model not fully replicating the metabolic features of natural chronological aging (Section 2.2), the relatively short 7-week induction period, and/or the possibility that serum PGC-1α does not track tissue-level (muscle or brain) expression of this fundamentally intracellular co-activator protein—a limitation we did not have the means to resolve with tissue-level assays in this study. Because tissue-level assays (e.g., Western blot, qPCR) of PGC-1α, SIRT2, or antioxidant enzymes were not performed, all mechanistic interpretations offered below should be read as hypotheses generated by, rather than confirmed by, the present serum-only data.

In young rats, PGC-1α did not differ significantly among exercise groups, though values were numerically higher in exercise groups than in the young control group (η^2^ = 0.038, small effect). In D-galactose-treated rats, moderate- and high-intensity exercise groups showed numerically higher PGC-1α than the control, which was again non-significant (*p* > 0.05, η^2^ = 0.075, medium effect). This numerical pattern is consistent with—though not direct evidence for—reports of exercise-induced mitochondrial biogenesis [37,38,39,40], whereas Carter et al. [38] reported statistically significant PGC-1α increases in aged animals under different conditions; differences may reflect our shorter 6-week exercise duration (versus 8–12 weeks in some comparator studies) or the serum-versus-tissue limitation noted above. Because we did not measure tissue PGC-1α, mitochondrial content, or biogenesis markers directly, we present this numerical pattern as a hypothesis consistent with our serum data rather than as a demonstrated mechanism.

Regarding MDA, moderate-intensity exercise was associated with significantly higher MDA in young rats (*p* < 0.05, η^2^ = 0.312, large effect), while high-intensity exercise showed the lowest values. This pattern is compatible with reports that moderate-intensity exercise can increase oxidative markers in some contexts [41,42] while stronger antioxidant-system activation may occur at higher intensities [43]. We did not measure antioxidant enzymes (e.g., catalase, superoxide dismutase, glutathione peroxidase) or total antioxidant capacity in this study, and these comparator studies used different models; therefore, this proposed mechanism cannot be established in our own animals and remains speculative. In D-galactose-treated rats, MDA differences were non-significant with a large effect size (*p* = 0.082, ε^2^ = 0.207), a pattern similar to the large-effect, non-significant findings reported by Gao et al. [44] and Li et al. [45]. Given the small sample size, this large point estimate should be interpreted as an imprecise estimate consistent with a range of true effects, including no effect, rather than as evidence of a probable but undetected effect; a larger study would be needed to distinguish these possibilities.

SIRT2, an NAD^+^-dependent deacetylase, plays a role in cellular metabolism, aging, and stress response. In young rats, SIRT2 differed significantly among groups overall (*p* < 0.05, ε^2^ = 0.265, large effect); after Bonferroni correction, only the high-intensity group differed significantly from the control, in a pattern broadly consistent with Brenmoehl et al. [46], while the numerically higher moderate-intensity value did not reach significance and should be treated as a non-significant trend. In D-galactose-treated rats, SIRT2 changes with exercise were non-significant (*p* = 0.096, ε^2^ = 0.199, large effect); as above, given the small sample size, this large-effect/non-significant combination should be read as an imprecise estimate rather than a confirmed absence of effect. We note that both PGC-1α and SIRT2 are intracellular proteins whose serum concentrations may not accurately track their expression in muscle or brain tissue—a limitation that applies across all of our biochemical findings and that we did not have the means to resolve with tissue-level assays (e.g., Western blot or qPCR on brain/muscle homogenate) in this study.

BDNF showed a numerically higher pattern with exercise in both young and D-galactose-treated rats, but the difference was not statistically significant in either group (*p* > 0.05, young: ε^2^ = 0.041; D-gal: ε^2^ = 0.096). We cannot determine from serum BDNF alone whether this reflects a true absence of central effect, insufficient sample size, or a mismatch between our 24 h post-exercise sampling time point and the time course of BDNF expression. We do not interpret the qualitative dentate gyrus observation (Section 3.3) as confirming a BDNF-mediated mechanism, since no BDNF immunostaining or tissue assay was performed.

Irisin did not differ significantly between groups in young (ε^2^ = 0.010) or D-galactose-treated rats (ε^2^ = 0.027), although all exercise groups showed numerically higher values. This is consistent with prior reports that short-duration exercise protocols may not significantly alter circulating irisin despite other neuroprotective associations reported in the literature [47,48].

The qualitative histological observation of greater dentate gyrus granule-cell density in exercised D-galactose-treated rats (Section 3.3) is, at most, hypothesis-generating. Young rats across all exercise groups maintained normal hippocampal architecture with no qualitative differences compared to young controls. In D-galactose-treated control rats, the dentate gyrus granular cell layer appeared visually thinner and more sparsely packed than in young controls. The exercised D-galactose-treated groups showed a qualitatively greater granule-cell density than D-galactose-treated controls, most visibly at high intensity. This directional pattern aligns with prior reports linking aerobic exercise to dentate gyrus granule-cell measures [49,50], but—as detailed in Section 2.5 and Section 3.3—it was not quantified, was assessed by a single rater, and was not accompanied by neurogenesis-specific markers (DCX, Ki67, BrdU, NeuN), synaptic markers (synaptophysin, PSD-95), or glial markers (GFAP, Iba1). We therefore explicitly do not interpret this finding as evidence of neurogenesis, regeneration, or synaptic plasticity, and recommend that any such claim await a dedicated follow-up study with stereological quantification and the tissue-level markers listed above.

This study has several limitations, consolidated here for clarity: (1) Only male rats were used, limiting generalizability to females. (2) Serum measurements of intracellular proteins (PGC-1α, SIRT2) and neurotrophic/myokine factors (BDNF, irisin) may not reflect tissue-specific (brain or muscle) expression; no tissue homogenate assays were performed. (3) The D-galactose model reproduces specific oxidative/histological features associated with aging but is not equivalent to natural chronological aging (Section 2.2); findings should not be extrapolated to naturally aged animals or to human aging without further confirmation. (4) Exercise intensity was assigned using literature-derived nominal categories based on fixed absolute treadmill speed, without individual physiological validation (VO_2_max, lactate, heart rate); the same absolute speed may have corresponded to different relative intensities in young versus D-galactose-treated animals (Section 2.3). (5) Histological analysis was based on H&E staining only and was qualitative, performed by a single blinded rater, without stereological quantification, neuronal-specific markers (e.g., NeuN), neurogenesis markers (e.g., DCX, Ki67, BrdU), glial markers (e.g., GFAP, Iba1), or molecular validation; the dentate gyrus finding should therefore be read as a preliminary, descriptive observation only, and is presented as a secondary, hypothesis-generating outcome rather than a central claim of this study (Section 2.5 and Section 3.3). Immunohistochemical and stereological analyses were not added retrospectively because they require processing of additional serial sections beyond the single representative section per animal collected under the original protocol; this is noted as a design limitation rather than an omission, and a dedicated follow-up study incorporating these markers from the outset is planned. (6) No behavioral testing (e.g., Morris water maze, novel object recognition) was performed, and because the animals were sacrificed at a single endpoint for tissue/serum collection, behavioral data cannot be added retrospectively; morphological observations therefore cannot be linked to any cognitive or functional outcome in this dataset. (7) Stress hormones (e.g., corticosterone) were not measured, so acute handling/sampling stress cannot be excluded as a contributor to biochemical variability. (8) A single post-exercise blood-sampling time point (24 h) was used, which may have missed peak expression of transiently regulated markers such as BDNF and irisin. (9) ELISA measurements were performed in singlicate rather than duplicate/triplicate due to serum volume constraints, so an experiment-specific intra-assay coefficient of variation could not be calculated; only the manufacturer’s kit-level CV is available. (10) Young control rats received no injection of any kind, whereas D-galactose-treated rats received daily intraperitoneal D-galactose injections; a vehicle (saline)-injected young control group was not included, so injection- and handling-related stress cannot be fully separated from the D-galactose effect itself. (11) Body weight was recorded at weeks 0, 3, and 6 and is reported in Table 4 (Section 3.2); food intake was not measured during the study and could not be used to help interpret the metabolic and oxidative-stress findings. (12) Treadmill incline was kept at 0% (flat) for all groups. (13) Sedentary control rats were not placed on the treadmill apparatus at any point; therefore, apparatus exposure, transport, and handling associated with the treadmill environment cannot be separated from the exercise effect itself in this study. (14) The effect size and other input parameters used in the original a priori G*Power sample-size calculation (Section 2.1) were not documented and cannot now be reliably reconstructed; the calculation should therefore be read as an approximate, non-reproducible historical record rather than a demonstrated guarantee of adequate power.

## 5. Conclusions

In young rats, six weeks of treadmill exercise was associated with a significant increase in SIRT2 (driven by the high-intensity group) and a significant, model-dependent change in MDA; PGC-1α and BDNF were numerically higher in the exercise groups but did not reach significance. In D-galactose-treated rats, no biochemical marker reached statistical significance; MDA and SIRT2 showed large effect sizes alongside non-significant *p*-values that, given the small sample size, should be read as imprecise estimates rather than evidence of a masked effect, warranting a larger follow-up study rather than a conclusion of ‘no effect’.

As a secondary, exploratory outcome, a qualitative, single-rater histological observation suggested greater dentate gyrus granule cell density in exercised D-galactose-treated rats; given the absence of quantification, stereology, and neurogenesis-specific markers, this should be read as hypothesis-generating rather than confirmed evidence of neuroregeneration. Overall, treadmill exercise produced statistically demonstrated, model-dependent biochemical changes in young rats (SIRT2, MDA, and a significant MDA model × exercise interaction). Findings in D-galactose-treated rats were inconclusive: MDA and SIRT2 showed larger point estimates but did not reach statistical significance and should not be interpreted as evidence of effect until confirmed in an adequately powered study. Because the D-galactose model does not fully replicate natural aging and exercise intensity was assigned nominally rather than physiologically validated, extrapolation to naturally aged animals or humans should await confirmatory studies with individualized intensity calibration, tissue-level assays, quantitative stereology, and behavioral testing.

## Figures and Tables

**Table 1:** Comparison of MDA and PGC-1α levels in rats.

Variables		Control	Low-Intensity Exercise	Moderate-Intensity Exercise	High-Intensity Exercise	*p*-Value
Young	MDA (ng/mL)	90.3 ± 10.4 ^b^	90.5 ± 11.5 ^ab^	103.2 ± 10.2 ^a^	85.9 ± 8.5 ^b^	0.013 *
	PGC-1α (pg/mL)	47.2 ± 11.4	54.1 ± 8.3	53 ± 14.6	48.5 ± 23.8	0.774
D-galactose-treated Groups	PGC-1α (pg/mL)	48.9 ± 17.6	44.7 ± 18.5	51.4 ± 13.1	57.8 ± 20.8	0.522

* *p* < 0.05. Different superscript letters in the same row indicate significant pairwise differences (*p* < 0.05); groups sharing a letter do not differ significantly. Malondialdehyde (MDA), Peroxisome Proliferator-Activated Receptor Gamma Coactivator 1-alpha (PGC-1α).

**Table 2:** Comparison of SIRT2, BDNF, irisin, and MDA levels in rats.

Variables		Control				Low-Intensity Exercise				Moderate-Intensity Exercise				High-Intensity Exercise				*p*-Value
		Median	P25	P75	IQR	Median	P25	P75	IQR	Median	P25	P75	IQR	Median	P25	P75	IQR	
Young	SIRT2 (ng/mL)	9.42	7.64	14.81	7.17	12.14	7.26	20.05	12.89	19.43	9.49	24.8	15.3	27.52	17.9	34.55	16.62	0.042 *
	BDNF (pg/mL)	94.48	62.9	165.4	102.47	134.75	80.44	201.9	121.5	132.03	99.2	143.4	44.23	118.3	61.6	201.2	139.59	0.735
	Irisin (ng/mL)	87.36	74.4	113.6	39.21	93.39	68.5	124.5	55.97	84.61	66.2	140.1	73.91	97.05	80.2	106.1	25.93	0.975
D-galactose-treated Groups	MDA (ng/mL)	90.88	85	107.5	22.49	86.86	77.79	94.77	16.98	81.16	75.1	86.25	11.18	86.05	81.1	88.78	7.71	0.082
	SIRT2 (ng/mL)	7.52	6.2	11.58	5.38	6.32	5.39	8.96	3.57	6.05	4.17	8.95	4.78	10.74	7.22	29.42	22.19	0.096
	BDNF (pg/mL)	79.92	62.5	187.7	125.15	136.2	100.3	177.4	77.03	90.77	53.3	135.8	82.52	92.38	59.2	153.8	94.60	0.399
	Irisin (ng/mL)	82.19	61.3	101.3	40.04	90.02	75.85	105.4	29.58	84.82	75.6	143.3	67.69	96.73	61.5	106.2	44.66	0.843

* *p* ˂ 0.05. Malondialdehyde (MDA), Sirtuin 2 (SIRT2), Brain-Derived Neurotrophic Factor (BDNF). The results are presented as median, 25th percentile (P25), 75th percentile (P75), and interquartile range (IQR).

**Table 3:** Shapiro–Wilk normality (within each model group) and Levene’s homogeneity-of-variance (across the eight model × exercise cells) test results.

Variable	Young W	Young *p*	Dgal W	Dgal *p*	Levene *p*
MDA	0.983	0.875	0.594	<0.001	0.345
SIRT2	0.896	0.005	0.679	<0.001	0.038
BDNF	0.556	<0.001	0.761	<0.001	0.464
PGC-1α	0.954	0.188	0.963	0.325	0.284
Irisin	0.890	0.004	0.458	<0.001	0.502

W = Shapiro–Wilk statistic; Levene *p* refers to the omnibus test for homogeneity of variance across all eight model × exercise cells. Body weight variables (weeks 0, 3, 6) were normally distributed in both models (all *p* > 0.05).

**Table 4:** Body weight (g, mean ± SD) at weeks 0, 3, and 6 by group.

Group	Week 0	Week 3	Week 6
Young Control	213.9 ± 23.5	224.6 ± 24.2	253.8 ± 21.6
Young Low-Intensity	245.8 ± 20.0	264.9 ± 19.5	304.1 ± 29.1
Young Moderate-Intensity	234.4 ± 14.4	253.9 ± 15.6	263.8 ± 11.9
Young High-Intensity	218.3 ± 14.9	213.1 ± 15.1	217.8 ± 15.0
D-galactose-treated Control	230.0 ± 6.4	245.4 ± 8.6	276.4 ± 11.3
D-galactose-treated Low-Intensity	233.5 ± 15.2	246.3 ± 16.0	276.1 ± 13.2
D-galactose-treated Moderate-Intensity	215.9 ± 15.0	233.9 ± 17.6	245.3 ± 17.9
D-galactose-treated High-Intensity	231.4 ± 16.8	226.0 ± 14.4	218.1 ± 14.0

## Data Availability

The original contributions presented in this study are included in this article. Further inquiries can be directed to the corresponding author.

## Abbreviations

The following abbreviations are used in this manuscript:

PGC-1α	Peroxisome Proliferator-Activated Receptor Gamma Coactivator 1-Alpha
MDA	Malondialdehyde
SIRT2	Sirtuin 2
BDNF	Brain-Derived Neurotrophic Factor
FNDC5	Fibronectin Type III Domain-Containing Protein 5
VO_2_max	Maximal Oxygen Consumption
DG	Dentate Gyrus
CA1–CA4	Cornu Ammonis Subfields 1–4
NAD^+^	Nicotinamide Adenine Dinucleotide
DCX	Doublecortin
Ki67	Antigen Kiel 67 (cell proliferation marker)
BrdU	Bromodeoxyuridine
NeuN	Neuronal Nuclei (neuronal marker)
GFAP	Glial Fibrillary Acidic Protein
Iba1	Ionized Calcium-Binding Adapter Molecule 1
PSD-95	Postsynaptic Density Protein 95
DNA	Deoxyribonucleic Acid
